# Gastroprotective effect of *Berberis vulgaris* on ethanol-induced gastric mucosal injury: Histopathological evaluations

**DOI:** 10.22038/AJP.2021.18113

**Published:** 2022

**Authors:** Marina Kapitonova, Sergey Gupalo, Renad Alyautdin, Ibrahim Abdel Aziz Ibrahim, Norita Salim, Azhar Ahmad, Saiful Bahri Talip, Tin Moe Nwe, Svetlana Morokhina

**Affiliations:** 1 *Faculty of Medicine and Health Sciences, UNIMAS, Kota Samarahan, Sarawak, Malaysia*; 2 *Faculty of Medicine MAHSA University, Bandar Saujana Putra, Jenjarom, Selangor, Malaysia*; 3 *Medicinal Products Safety, Scientific Centre for Expert Evaluation of Medicinal Products, Moscow, Russia*; 4 *Department of Pharmacology, Sechenov University, Moscow, Russia*; 5 *Department of Pharmacology and Toxicology, Faculty of Medicine, Umm Al-Qura University, Makkah, Saudi Arabia*; 6 *Institute of Medical Molecular Biotechnology, and Faculty of Medicine, UiTM, Sungai Buloh, Selangor, Malaysia*; 7 *Department of Pharmacognosy, Sechenov University, Moscow, Russia*

**Keywords:** Berberis vulgaris, Stomach ulcer, Prostaglandin E2

## Abstract

**Objective:**

Modern treatment of peptic ulcers includes antibacterial and gastroprotective medications. However, current anti-ulcer drugs possess severe side effects. Therefore, all attempts to find new effective medications free from side effects are justified. Though *Berberis vulgaris* is a medicinal plant commonly used for the treatment of numerous disorders, gastroprotective effect of its leaf extract was not investigated before.

**Materials and Methods:**

Gastric ulcer was modelled in Sprague-Dawley rats after treatment with *B. vulgaris* leaf extract containing 0.07% of alkaloids, 0.48% of flavonoids and 8.05% of tanning substances, 10 or 50 mg of dry extract/kg, changes in the stomach mucosa were assessed semi-quantitatively, and the gastric wall was evaluated for prostaglandin E2 level using ELISA and assessed histologically by calculation of the lesion index.

**Results:**

*B. vulgaris* leaf extract at the dose of 50 mg/kg reduced the macroscopic ulcer score and the microscopic lesion index, increased prostaglandin E2 concentration in the gastric wall significantly higher than atropine and *B. vulgaris* leaf extract 10 mg/kg.

**Conclusion:**

The gastroprotective effect of the high dose of *B. vulgaris* leaf extract may be due to stimulation of prostaglandin E2 secretion in the stomach, and anti-oxidative and anti-inflammatory properties of polyphenolic complex of flavonoids and tannins present in the leaves of this plant.

## Introduction

Gastric ulcer is one of the most common diseases causing life-threatening complications, such as perforation of the gastric wall and bleeding. In addition, gastric ulcer reduces the quality of life of patients and is a potential risk of cancer (Komar et al., 2018; Yegen, 2018; Kavitt et al., 2019; Tarasconi et al., 2020). The etiological factor in the development of gastric ulcer is *Helicobacter pylori* (*H. *pylori), whose incidence in developed countries reaches 10% (Quach et al., 2018). Irritation of the gastric mucosa and cholinergic activation caused by smoking, hydrochloric acid, pepsin, ischemia, nonsteroidal anti-inflammatory drugs (NSAIDs), hypoxia, alcohol, etc. may damage the mucosal barrier and provoke ulceration. Many other mechanisms have been implicated in the pathogenesis of gastric ulcers, including inhibition of prostaglandin synthesis and cell proliferation, and changes in gastric blood flow, and gastric motility (Cryer, 2001; Toma et al., 2005; Tytgat**,** 2011; Tarasconi et al., 2020).

Modern therapy of gastric ulcers includes antimicrobial treatment and protection of the gastric mucosa. However, the development of *H. pylori*, resistance significantly reduces the effectiveness of antibiotic treatment (Ahmad et al., 2019; Suzuki et al., 2019). Currently used anti-ulcer agents, such as H_2_ antagonists, proton pump inhibitors, prostaglandin analogs, and cholinoblockers, have significant side effects. Moreover, recently, serious problems were shown for H_2_ antagonists, which were considered to be among the most effective anti-ulcer medications, as a toxic impurity was found in ranitidine drug products. In April 2020, the FDA requested to withdraw ranitidine from the market in the United States, since a contaminant known as N-Nitrosodimethylamine (NDMA) was determined in ranitidine products during storage, including home storage, at unacceptable levels (FDA, 2020). Thus, the problem of gastric ulcer treatment, which seemed to be almost solved, becomes relevant again, and attempts to discover new drugs with lower cost and fewer side effects need to be undertaken (Al-Sayed et al., 2020). 

Natural products are an essential resource for the prevention and treatment of gastric ulcers (Berłowski et al., 2013; Ahmad et al., 2019; Al-Sayed et al., 2020). Barberry (*Berberis vulgaris L.,* Var. asperma Don., family Berberidaceae) is traditionally cultivated in Iran (especially in Birjand and Qaen) where it is known as "zereshk"; it also grows in the south of Europe and northeast of the USA; it has been widely used in traditional medicine for the treatment of cardiovascular and metabolism disorders (El-Wahab et al., 2013; Tabeshpour et al., 2017; Asadi et al., 2019; Imenshahidi and Hosseinzadeh, 2019). Medicinal properties for all parts of the plant have been reported, including antimicrobial, antioxidant, anti-inflammatory and anticholinergic actions (Mokhber-Dezfuli et al., 2014; Abushouk et al., 2917; Rahimi-Madiseh et al., 2017; Mirhadi et al., 2018; Kalmarzi et al., 2019**)**. This plant has been for a long time used for the treatment of gastrointestinal disorders (Majeed et al., 2015; Zarei et al., 2015; Imenshahidi and Hosseinzadeh, 2016)**.** Barberry fruit extract showed antioxidant properties and an inhibitory activity on *Helicobacter pylori *growth; its gastroprotective effect was demonstrated to be mediated through modulation of nitric oxide synthase (iNOS) gene expression: activation of endothelial nitric oxide synthase (eNOS) and inhibition of inducible nitric oxide synthase (iNOS) (Fazaei et al., 2013). To the best of our knowledge, so far, no studies have been done to investigate the effect of *B. vulgaris* leaf extraction (BV) on gastric ulcer. Therefore, we have undertaken this study to evaluate the gastroprotective effect of BV on ethanol-induced gastric mucosal injury in rats.

## Materials and Methods

**Material**

*Berberis vulgaris *leaves were harvested in the Botanic garden of the Sechenov University (Moscow, Russia, 55°44'46.615" N 37°31'48.886" E) in summer 2014 in a phase of full development. Plant samples were identified at the Department of Pharmacognosy of the Pharmaceutical Faculty of the Sechenov University and placed in the department herbarium (herbarium number: 00725/150614)*. *Their 1:10 extract was prepared using 70% alcohol using percolation and consequent sedimentation during 48 hr at 10°C. The extract obtained met all the requirements of the Russian State Pharmacopea XIII. The presence of the biologically active substances in the extract of the *B. vulgaris* leaves was confirmed using qualitative reactions. Alkaloids were detected using Dragendorff's reagent producing orange-red precipitate, flavonoids - using 1% aluminum chloride in 95% alcohol, producing yellow-green staining. At the same time, tanning substances were determined using ferric ammonium sulfate, giving blue-green color.

Spectrophotometrically alkaloids content in the extract was determined as 0.070±0.018% and flavonoids as 0.48±0.09%, while tanning substances content was estimated as 8.05±0.17% by titrating the extract with standard potassium permanganate solution.

**Animals**

A total of 30 Sprague-Dawley rats weighing 200-250 g were involved in the study. They were divided into 5 groups with 6 rats per group: 3 control and 2 experimental groups. The 1^st^ group (N) and 2^nd^ group (A) of negative control received normal saline, the 3^rd ^group of positive control (A+At) – 50 mg/kg of atropine, the 4^th^ group (A+b) – BV (10 mg of dry extract/kg), and the 5^th^ group (A+B) - BV (50 mg/kg). After 30 min, animals of the N group received normal saline, while the rest of the groups received absolute alcohol (0.5 ml/kg of weight) to induce stomach ulcer as recommended (Pan et al., 2005). After 1 hr, animals were sacrificed under ketamine anaesthesia. The stomach was removed, the content was tested for pH, the organ was cut along the greater curvature, rinsed with distilled water, spread on the pad using needles, and assessed visually for the depth of the lesions. Scoring of the ulcer was made as follows: normal coloured stomach (0), red coloration (0.5), spot ulcer (1), hemorrhagic streak (1.5), deep ulcers (2), perforation (3) as described earlier (Dashputre and Naikwade, 2011). The anterior wall of the stomach was sampled for ELISA (PgE_2_), and the posterior wall was fixed in formalin and processed for histological evaluation of the alcohol-induced lesions. The histological slides 3 μm thick were stained with hematoxylin-eosin and assessed independently by two researchers semi-quantitatively for the microscopic lesions. 

**Reagents and equipment**

The following chemicals and equipment were used in the study:

**- **Phosphate Buffered Solution (PBS), Sigma Aldrich

**-** Lysis Buffer 17 (R&D system, Abingdon, UK, catalog #895943)

**-** Microplate reader capable of measuring absorbance at 450 nm, (Victor X, Perkin Elmer, USA)

**-** Tissue homogenizer, (Omni-Ruptor 4000, OMNI International Inc., US)

**-** Horizontal orbital shaker with the speed of 500±50 rpm (VISION Scientific Co. Ltd, Korea).

**Methods**

In our research, pH of the gastric content was tested using litmus paper (More et al., 1983). Macroscopic evaluation of ulcer scoring was done as recommended (Dashputre and Naikwade, 2011). 

For ELISA, 100 mg of rat stomach tissue was washed with 0.1 M phosphate buffer and lysed in 1 ml of homogenization buffer (0.1 M phosphate buffer, pH 7.4 containing 1 mM EDTA and 10 mm indomethacin) using tissue homogenizer. Then, samples lysate was centrifuged at 8,000 x g for 10 min to pellet particulate matter. The supernatant was transferred into a clean 1.5 ml tube. Prostaglandin E2 concentration in samples was assayed using the Prostaglandin E2 express EIA kit (Caymanchem). Data analysis was done using spreadsheets available at www.caymanchem.com/analysis/eia according to the manufacturer protocol using microplate reader VictorTM X, Perkin Elmer, US, at 450 nm wavelength. The optical density reading of the sample at 450 nm wavelength was subtracted with 570 nm wavelength to avoid imperfection plate. The results of the ELISA are expressed as pg/mL. The lesion index was estimated by two independent researchers as recommended (Nur Azlina et al., 2013). 

**Statistical analysis**

All data are presented as the mean±SEM, with n=6 per group. The results were analyzed by one-way analysis of variance (ANOVA) followed by Student–Newman–Keuls multiple comparison test; p*<*0.05 values were considered statistically significant.

## Result

**Determination of the pH of the gastric content**

**Figure 1:**
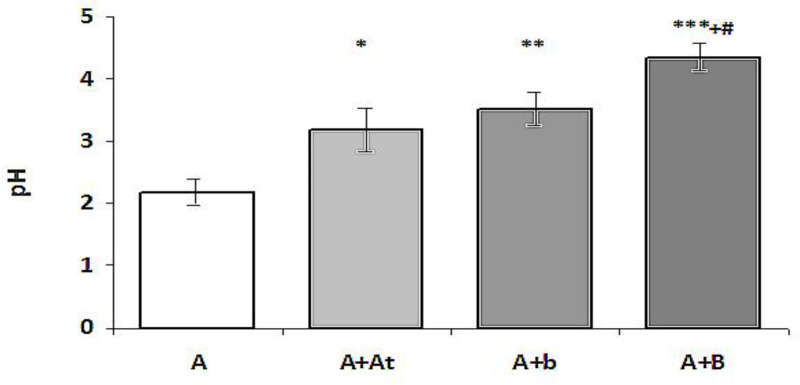
pH of the gastric content in study rats (Mean±SEM)

The results are shown in Figure1. As follows from Figure1, the pH of the gastric content in the groups A+At and A+b was significantly higher compared to the group A (p<0.05 and p<0.01, respectively). There was no significant difference in the pH between the groups A+At and A+b (p>0.05), while in the group A+B, it was significantly higher than all the other groups (p<0.001 compared to the group A and p<0.05 compared to the groups A+At and A+b).

**Macroscopic evaluation**

**Figure 2:**
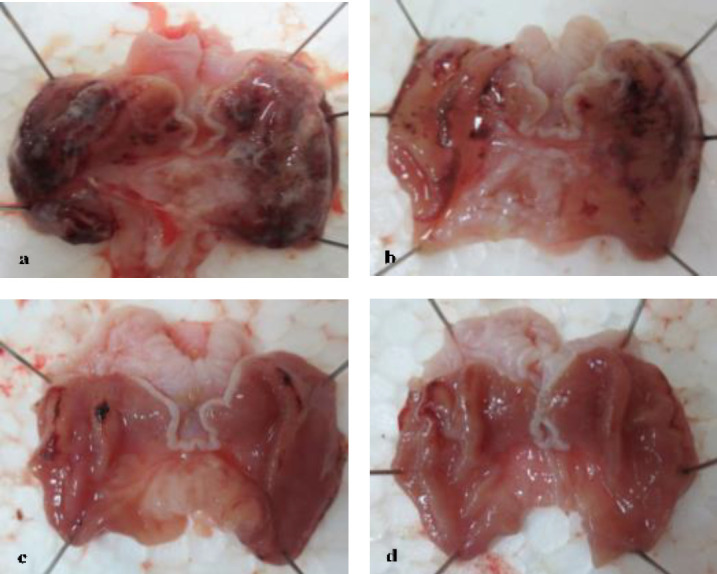
The gross appearance of gastric mucosa in the control and experimental rats

The study revealed a significant damaging effect of ethanol on gastric mucosa (Figure 2a). At the same time, the animals which received only normal saline showed no changes on the inner surface of the stomach. The animals that received ethanol (group A) developed a considerable macroscopic mucosal damage evidenced by the presence of extensive ulcerations and haemorrhages (Figure 2b) covering more than 80% of the gastric mucosa. The area and intensity were somewhat reduced by the administration of atropine (the group A+At) with a decreased area of gastric mucosal necrosis, erosions, haemorrhages, and hyperemia (Figure 2c) covering more than 50% of the gastric mucosa.

A-alcohol (negative control) *p<0.05 compared to A

A+At-alcohol+atropine **p<0.01 compared to A

A+b-alcohol+BV (10 mg) ***p<0.001 compared to A

A+B-alcohol+BV (50 mg) +p<0.05 compared to A+At. #p<0.05 compared to A+b

However, combined administration of ethanol with BV showed macroscopic toxicity attenuation, preserving the most of morphological integrity of the gastric mucosa with the presence of gastric erosions and haemorrhages covering up to 30% in the group A+b and generalized erythema with occasional mucosal haemorrhages seen in the animals of the group A+B for 10 mg/kg and 50 mg/kg of BV. 

**Figure 3:**
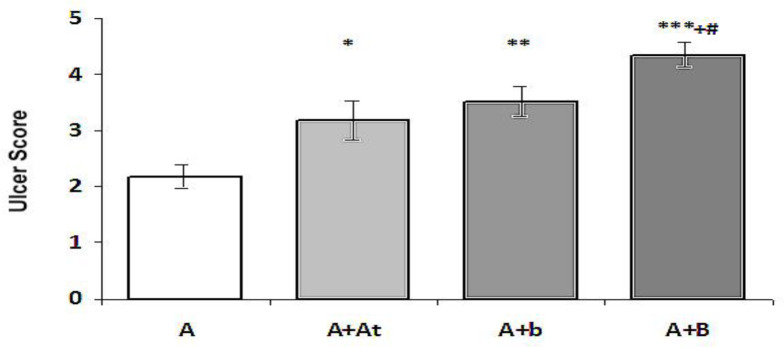
Ulcer scoring in the study rats (Mean±SEM)

The results of ulcer scoring are shown in Figure 3. As shown in Figure 3, the ulcer score was reduced in the A+At group compared to the A group, but this reduction was not significant (p>0.05). It was significantly (p<0.05) and highly significantly (p<0.001) reduced in the A+b and A+B groups compared to the A group respectively; moreover, in the group A+B, it was significantly reduced compared to the groups A+At (p<0.001) and A+b (p<0.05). In the group A+b, the ulcer score was lower compared to the group A+At, but this difference was not significant (p>0.05).

**Measurement of the content of prostaglandin E2 in the gastric wall using ELISA**

**Figure 4:**
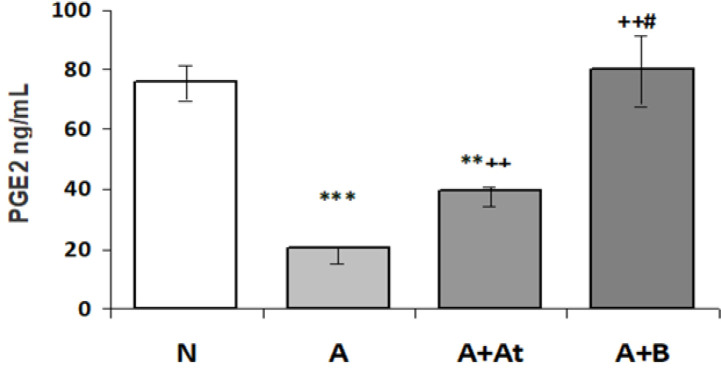
Prostaglandin E_2_ content (ng/ml) in the wall of the stomach of the study rats (Mean±SEM)

As shown in Figure 4, the level of PGE2 in the wall of animals from the group A was highly significantly reduced compared to the normal rats (p<0.001). In group A+At, it was significantly higher compared to the group A (p<0.01), but it was significantly lower than that of the normal rats (p<0.01). In the group A+B, it was significantly higher than the group A (p<0.01) and group A+At (p<0.05), while it was slightly higher without reaching the level of significance (p>0.05) than the normal rats.

a) Ethanol-treated group (Group A): severe injuries to the stomach mucosa with diffuse hyperemia, multiple petechiae, and slouched-off superficial gastric mucosa, especially at the top of the gastric folds, revealing multiple erosions covered with whitish slough.

b) Atropine-treated group (Group A+At): less severe necroerosive lesions within the gastric mucosa revealing focal hyperaemia with swollen folds and superficial erosions.

c) BV-treated group (Group A+b, 10 mg/kg): moderate focal haemorrhages and disruptions of the surface lining of the stomach.

d) BV-treated group (Group A+B, 50 mg/kg): mild hyperaemia and minimal focal haemorrhages with mild disruptions of the surface lining in the gastric mucosa and slightly swollen mucosal folds.

A-alcohol (negative control) and *p<0.05 compared to the group A

A+At-alcohol+atropine ***p<0.001 compared to the group A

A+b-alcohol+BV (10 mg) +++p<0.001 compared to the group A+At

A+B-alcohol+BV (50 mg) #p<0.05 compared to the group A

**Histopathological study**

 Histopathological study of gastric lesions in the ethanol-treated group (group A) displayed marked changes in gastric mucosa and submucosa including severe necrosis and desquamation of mucosal epithelial cell, vacuolization, oedema and some dilation of gastric glands along with infiltration of inflammatory cells (neutrophils), haemorrhage in lamina propria, degenerative changes in gastric glands, congestion of blood and oedema in submucosal layer with scattered inflammatory cells, mostly neutrophils.

**Figure 5:**
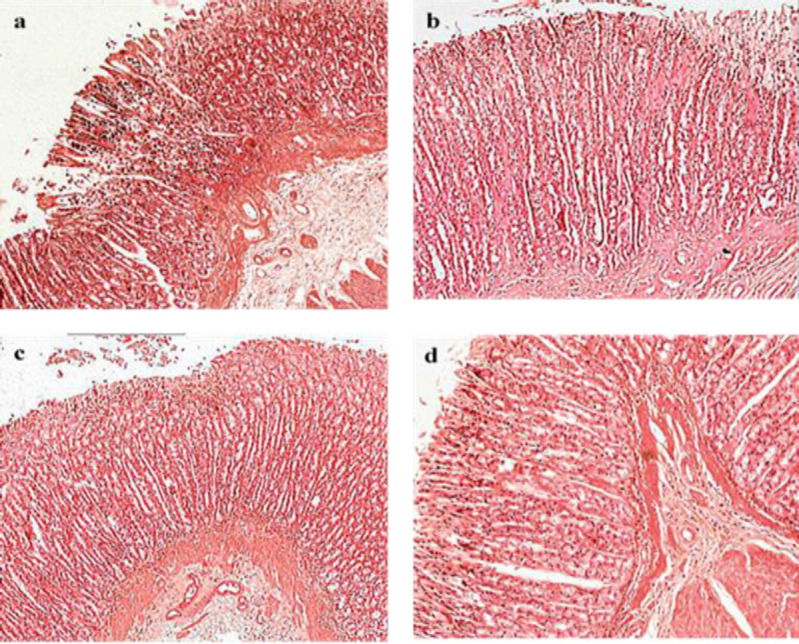
Microphotographs of the H&E stained histological slides of the stomach of the control and experimental rats. x100. a) Group A: marked changes in gastric mucosa and submucosa including severe necrosis and sloughing of the superficial mucosal epithelium with the formation of mucosal erosions, oedema with infiltration of inflammatory cells (neutrophils), haemorrhages in lamina propria, degenerative changes in gastric glands; in the submucosal layer: congestion of blood and oedema with scattered inflammatory cells, mostly neutrophils. b) Group A+At: less pronounced gastric damage compared to the Group A; the gastric mucosa exhibited focal necrosis with sloughed off superficial mucosal epithelium and formation of superficial focal erosions limited to the mucosa with scattered inflammatory infiltration predominantly with neutrophils, whereas in other areas the gastric glands were almost normal in appearance; mild submucosal oedema with scattered neutrophil infiltration. c) Group A+b: gastric lesions, characterized by focal areas of mild epithelial cells disruption and moderate mucosal oedema. Scattered neutrophils in submucosa were observed. d) Group A+B: almost normal epithelial lining with some focal superficial epithelial denudation

 Pre-treatment with atropine (group A+At) demonstrated less pronounced gastric damage compared to the ulcer-negative control (group A). The gastric mucosa exhibited focal necrosis limited to mucosa only, whereas, in other areas, the gastric glands were almost normal in appearance. There was mild submucosal oedema with scattered neutrophilic infiltration. Pre-treatment with BV (10 mg/kg, group A+b) resulted in gastric lesions, characterized by focal areas of mild sloughing off of the epithelial cells and moderate mucosal oedema. Scattered neutrophils in submucosa were observed. However, in the group treated with a higher dose of BV (50 mg/kg, group A+B), there was minimal sloughing of epithelial cells. Hence, the epithelium of gastric mucosa can be considered to be almost intact (Figure 5a, b, c, and d).

**Figure 6:**
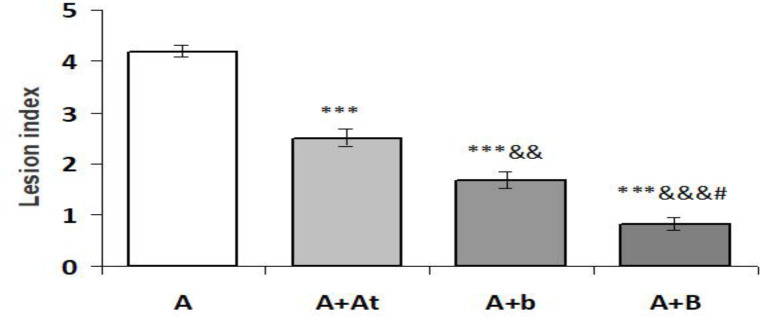
Lesion index in the stomach of study rats (Mean±SEM).

The results of the estimation of the microscopic lesion index are shown in Figure 6. Lesion index was highly significantly reduced in the group A+At compared to the group A (p<0.001); it was also highly significantly reduced in the group A+b compared to the group A (p<0.001) and group A+At (p<0.01); and it was highly significantly reduced in the group A+B compared to the groups A (p<0.001) and A+At (p<0.001), and significantly reduced compared to the group A+b (p<0.05).

Overall, the results of the experiment demonstrated that treatment with atropine significantly increased the pH of the gastric juice, while treatment with BV caused its highly significant increase, which was more prominent in the A+B experimental group. The effect of BV was dose-dependent, and the difference between A+b and A+B groups was significant. Atropine did not cause any significant decrease of the ulcer score, while this parameter was significantly decreased in the A+b group, and highly significant in the A+B group. The difference in the ulcer score between the A+B group and other treated groups was also significant. PGE2 content in the animals with untreated ulcers was significantly lower than in normal rats.

N–normal rats, **p<0.01 compared to the group N

A–alcohol (negative control); ***p<0.001 compared to the group N

A+At– alcohol+atropine; **++**p<0.01 compared to the group A

A+B– alcohol+BV (50 mg)** #**p<0.05 compared to the group A+At

Treatment with atropine increased PGE2 content compared to untreated animals, but it was still much less than that of the normal animals. Treatment with BV 50 mg increased PGE2 to the normal level, making it highly significantly larger than in untreated and atropine-treated groups. The microscopic lesion index proved to be the most sensitive parameter of our study. It was highly significantly decreased in all treatment groups, while in the A+b group it was significantly lower than the A+At group; and in the A+B group; it was significantly lower than in both A+At and A+b groups.

## Discussion

Despite the availability of highly effective drugs for the treatment of gastric ulcers, the prevalence of this disease remains high, and it continues to be a course 

of significant morbidity and mortality worldwide, therefore proper management is important for the minimization of serious complications (Komar et al., 2018; Narayanan et al., 2018; Roberts-Thomson, 2018; Kavitt et al., 2019). The need to search for new alternative medications for the treatment of gastric ulcer is dictated by a number of factors. Currently, in the treatment of gastric ulcers, the drugs of choice are proton pump inhibitors (PPIs), which irreversibly inhibit H+K+ATPase in the parietal cells of the stomach and thereby reduce the secretion of hydrochloric acid.

A–alcohol (negative control) ***p<0.001 compared to the group A

A+At–alcohol+atropine **++**p<0.01 compared to the group A+At

A+b–alcohol+BV (10 mg) **+++**p<0.001 compared to the group A+At

A+B–alcohol+BV (50 mg)** #**p<0.0 compared to the group A+b

The high efficiency of PPIs creates the impression that there are no vacant niches in this segment of the pharmaceutical market (Halfdanarson et al., 2018). While the advantages of short-term PPIs administration are well documented, the consequences of long-term deep inhibition of hydrochloric acid secretion are to be further elucidated. It is known that there is an increased risk of neuroendocrine tumors and carcinoma with prolonged use of PPIs. Enterochromaffin cells are a target for gastrin, and hyperstimulation of these cells can lead to carcinogenesis in PPIs users (Tang et al., 2013). In addition, PPIs use is associated with a 1.76-fold increase in the risk of chronic kidney disease, which may be related to the blockage of kidney transport systems (Lazarus et al., 2016). Recent studies have found that long-term PPIs use is also associated with the development of dementia and Alzheimer's disease (Ortiz-Guerrero et al., 2018; Cooksey et al., 2020). Profound and long-lasting inhibition of gastric acid secretion leads to disturbances in the absorption of vitamin B12, calcium, magnesium and iron, development of osteoporosis and, consequently, to an increased risk of bone fractures (Ajmera et al., 2012; Eusebi et al., 2017; Freedberg et al., 2017; Ueberschaer and Allescher, 2017; Miller, 2018; Nassar and Richter, 2018). Along with this, it must be borne in mind that CYP2C19 genetic polymorphism, which affects the activity of PPIs, is widespread in the world, especially in Asia, where the incidence reaches 15-20% (Tang et al., 2013). This leads to a 5-10 time increase in the concentration of PPIs in the blood of poor metabolizers. These problems caused by the long-term use of PPIs necessitate the search for other options, fundamentally new drugs for the treatment of gastric ulcers (Bhatia et al., 2015; Minaiyan et al., 2018; Boushra et al., 2019; Raeesi et al., 2019).

At the same time, phenolic compounds have been shown to play an essential role in the prevention of gastric ulceration (Sumbul et al., 2011; Farzaei et al., 2015; Gupta et al., 2020). In our experimental work, we chose the ethanol-induced model of the gastric ulcer, which is the most common animal model for the study of new anti-ulcer drugs (Jabri et al., 2017; Karampour et al., 2019). Alcohol consumption is known as one of the risk factors for gastric ulceration (Sumbul et al., 2011; Lv et al., 2019). The pathophysiological background of this model is related to the ethanol-induced necrotic changed of the gastric mucosa with subsequent infiltration of the inflammatory cells, and reduction of secretion of bicarbonates, gastric mucus, and nitric oxide. In addition, ethanol reduces gastric blood flow and causes oxidative stress, increasing the production of malondialdehyde and reducing the production of glutathione (Alvarez-Suarez et al., 2011; Arab et al., 2015, Al-Quraishy et al., 2017)**.** It was shown that the antioxidant activity of the alcohol extract of barberry leaves is due to the presence of polyphenols in it (Berłowski et al., 2013), though its gastroprotective effect in peptic ulcer was not assessed before (Majeed et al., 2015). An *in vitro* study demonstrated the antioxidative cytoprotective effect of *Berberis aristata* on the gastric epithelial cells co-cultured with *H. pylori* (Zaidi et al., 2012). A few investigations demonstrated the gastroprotective effect of extracts of different parts of *Berberis* plants in peptic ulcer and other diseases of the gastrointestinal tract, but most of them used fruits and seeds, while leaves mainly remain unattended (Farzaei et al., 2013; Majeed et al., 2015; Rahimi-Madiseh et al., 2017; Alamzeb et al., 2018 Kalmarzi et al., 2019)

Our results confirmed data of other authors (Majeed et al., 2015; Kalmarzi et al., 2019) regarding the gastroprotective effect of *B. vulgaris* through its astringent and local anti-inflammatory action and complemented it with new information regarding stimulation of PGE2 secretion by gastric mucosa which proved to be an additional powerful protective factor which was not described before in research on defensive mechanisms of this plant against ulcerogenic agents.

The antioxidant and anti-inflammatory effect of BV in our model of a gastric ulcer, allowed us to preserve the cells of the gastric mucosa from ethanol-induced damage and maintain its functional activity in relation to the production of protective gastric mucus and prostaglandins (Farzaei et al., 2015). This protective effect can explain the high content of prostaglandin E2 in the mucosa determined in our experiments. 

Obviously, this effect is not associated with an action of BV on the acidity of the gastric secretions, since atropine, which reduces the secretion of hydrochloric acid, did not significantly affect the content of prostaglandins in the gastric mucosa. Our results correlate with the previous findings (Berenguer et al., 2006) regarding prostaglandin-associated gastroprotective effect in modelled peptic ulcer of another plant (*Rhizophora mangle* *L*.) known for high content of tannins. Our results do not support the statement (Kupeli et al., 2002; Yesilada and Kupeli, 2002; Rad et al., 2017) regarding the provocation of gastrointestinal ulceration by the *Berberis* *crataegina* root extract. This contradiction may be explained by the application of root rather than leave extract in these studies and their use of another *Berberis* species (*Berberis crataegina DC*). In these papers, the ulceration effect of *Berberis crataegina* *DC* root extract is referred to the presence of alkaloids, mainly berberine in it, while in our study the content of alkaloids in BV was low, and we associate its gastroprotective effect with tannins and flavonoids present in it at much higher concentrations. 

The results of the present study strongly indicate the gastroprotective effects of BV in experimental ethanol-induced gastric ulcer. The ameliorating effect of ethanolic extract of the *B. vulgaris *leaves on the gastric ulcer might be assigned to the observed prostaglandin-associated anti-oxidative and anti-inflammatory properties of the polyphenolic complex of flavonoids and tannins present in BV. It is well known that flavonoids show anti-ulcer and anti-inflammatory activities, and most of the flavonoids are potent antioxidants. Tannins exhibit a proven anti-inflammatory, antimicrobial, and astringent action. Both tannins and flavonoids are known to increase secretion of prostaglandins in the gastric mucosa which exhibit accelerated reparative processes and its strengthened defensive potential. However, further studies are required to examine the anti-ulcer efficacy of BV in a clinical setup. 

## Conflicts of interest

The authors have declared that there is no conflict of interest.
